# Dyke-Davidoff-Masson syndrome in a young adult: Lifelong hemiparesis and characteristic MRI findings

**DOI:** 10.1016/j.radcr.2026.04.010

**Published:** 2026-05-07

**Authors:** Yohannes Girma Zewdie, Chalew Abebe Mengesha, Hana Yeshewas Genetirune

**Affiliations:** a Department of Radiology, Addis Ababa University, Addis Ababa, Ethiopia; b Department of Radiology, Gonder University, Gonder, Ethiopia; c Anesthesia Department, Jimma university, Jimma, Ethiopia

**Keywords:** Dyke-Davidoff-Masson syndrome, Cerebral hemiatrophy, Hemiparesis, MRI, Wallerian degeneration, Calvarial thickening

## Abstract

Dyke-Davidoff-Masson syndrome (DDMS) is a rare disorder characterized by cerebral hemiatrophy and compensatory calvarial changes. We report a 22-year-old male with lifelong left-sided spastic hemiparesis and developmental delay, with a history of prematurity, neonatal meningitis, and childhood head trauma. Brain MRI demonstrated right cerebral hemiatrophy, exvacuo dilatation of the lateral ventricle, ipsilateral sulcal prominence, calvarial thickening, frontal sinus enlargement, and Wallerian degeneration of the corticospinal tract. This case is notable for the coexistence of multiple early-life insults contributing to acquired DDMS and the clear demonstration of classic imaging findings in adulthood. The report highlights the importance of correlating clinical history with imaging for accurate diagnosis and long-term management.

## Introduction

Dyke-Davidoff-Masson syndrome (DDMS) is a rare neurological condition characterized by cerebral hemiatrophy, ipsilateral calvarial thickening, and hyperpneumatization of the frontal and ethmoid sinuses [1,2]. It results from congenital or acquired brain insults occurring during the prenatal, perinatal, or early childhood period. Although its exact prevalence is unknown, DDMS is considered an uncommon entity, with most data derived from case reports and small case series [[3], [4], [5]]. Clinically, patients commonly present with contralateral hemiparesis, seizures, facial asymmetry, and varying degrees of cognitive impairment and developmental delay [3,4,6]. Imaging plays a crucial role in diagnosis and in distinguishing congenital from acquired causes.

## Case presentation

A 22-year-old male presented with lifelong left-sided weakness and spasticity, along with a history of developmental delay, including speech impairment. He was born preterm at 33 weeks of gestation via cesarean section due to prolonged labor, with no documented antenatal care.

During the neonatal period, he developed meningitis requiring prolonged hospitalization in the neonatal intensive care unit for over one month. At the age of five years, he sustained head trauma complicated by a right-sided subdural hematoma, which was surgically evacuated.

At presentation, he complained of persistent joint stiffness and chronic weakness on the left side. Neurological examination revealed left-sided spastic hemiparesis with increased muscle tone, hyperreflexia, and mild contractures involving both upper and lower limbs. Speech was delayed, consistent with his developmental history.

## Imaging findings

**Fig. 1:**
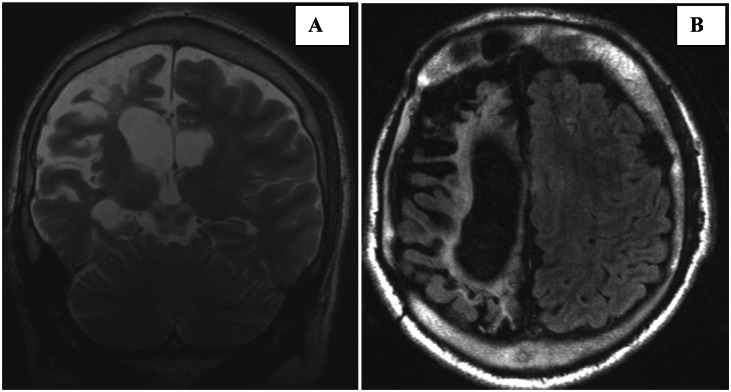
(A) Coronal T2-weighted MRI demonstrates right cerebral hemiatrophy with ipsilateral sulcal prominence and ex-vacuo dilatation of the lateral ventricle. (B) Axial FLAIR image shows dilatation of the right lateral ventricle with periventricular T2/FLAIR hyperintensity, consistent with gliotic changes.

**Fig. 2:**
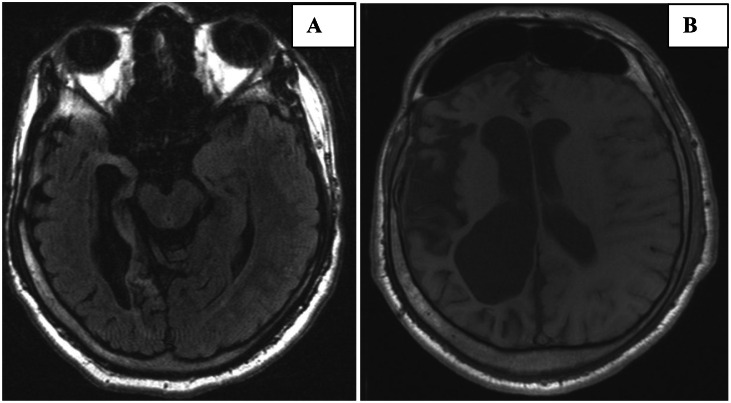
(A) Axial FLAIR image demonstrates asymmetric atrophy of the right cerebral peduncle, consistent with Wallerian degeneration. (B) Axial T1-weighted MRI shows prominence of the right frontal sinus with asymmetric thickening of the right calvarium.

Brain MRI revealed marked right cerebral hemiatrophy with associated ex-vacuo dilatation of the right lateral ventricle (Fig 1A). There was prominence of the ipsilateral cortical sulci, reflecting volume loss. The right calvarium showed compensatory thickening, along with enlargement of the ipsilateral frontal sinus (Fig. 2B).

Additionally, periventricular and deep white matter changes consistent with gliosis were observed (Fig. 1B). Wallerian degeneration along the right corticospinal tract was evident, indicating chronic neuronal injury (Fig. 2A). Gray-white matter differentiation was preserved.

These findings are characteristic of DDMS and support a longstanding insult to the right cerebral hemisphere [[7], [8], [9]].

## Discussion

DDMS is broadly classified into congenital (primary) and acquired (secondary) forms. Congenital cases typically result from intrauterine vascular insults or infections, whereas acquired cases arise from early childhood brain injury such as trauma, infection, or ischemia [2,3,10].

The present case is most consistent with acquired DDMS, given the history of prematurity, neonatal meningitis, prolonged neonatal intensive care admission, and subsequent childhood head trauma with subdural hematoma. The coexistence of multiple early-life insults makes this case distinctive and highlights the cumulative impact of repeated neurological injury on cerebral development.

Radiologically, DDMS is characterized by cerebral hemiatrophy, compensatory ventricular dilatation, ipsilateral calvarial thickening, and hyperpneumatization of paranasal sinuses [[7], [8], [9]]. MRI is particularly valuable in demonstrating white matter gliosis, cortical atrophy, and Wallerian degeneration, which may not be fully appreciated on CT imaging [7,8].

Although DDMS is typically diagnosed in childhood, cases presenting in adulthood have been reported, often due to delayed evaluation or milder early symptoms [4,9]. This underscores the importance of recognizing characteristic imaging findings even later in life.

Differential diagnoses include Rasmussen encephalitis, which presents with progressive neurological deterioration and refractory seizures without calvarial changes; Sturge-Weber syndrome, characterized by leptomeningeal angiomas and cortical calcifications; post-stroke hemispheric atrophy, which lacks compensatory osseous changes when occurring later in life; and hemimegalencephaly, which demonstrates enlargement rather than atrophy of the affected hemisphere [3,8,5]. The presence of compensatory calvarial thickening and sinus enlargement in this case strongly supports the diagnosis of DDMS.

Management is primarily supportive and includes physiotherapy for motor deficits, speech therapy, and seizure control when indicated. Early recognition is important to optimize rehabilitation and minimize long-term disability [6,3].

## Conclusion

Dyke-Davidoff-Masson syndrome is a rare but important cause of lifelong hemiparesis and developmental delay, most often resulting from perinatal or early childhood brain injury. This case highlights the contribution of multiple early-life insults in acquired DDMS and demonstrates classic imaging findings persisting into adulthood. Recognition of this entity is essential for accurate diagnosis, appropriate rehabilitation, and long-term patient management.

## Patient consent

Both oral and written informed consent were obtained from the patient for publication of this case report and the associated images.
